# Developing a Delphi-Based Comprehensive Core Set from the International Classification of Functioning, Disability, and Health Framework for the Rehabilitation of Patients with Burn Injuries

**DOI:** 10.3390/ijerph18083970

**Published:** 2021-04-09

**Authors:** Yu-Ru Lin, Jr-Yi Wang, Shun-Cheng Chang, Kwang-Hwa Chang, Hung-Chou Chen, Reuben Escorpizo, Shih-Wei Huang, Tsan-Hon Liou

**Affiliations:** 1 Department of Physical Medicine and Rehabilitation, Shuang Ho Hospital, Taipei Medical University, Taipei 23561, Taiwan; 14001@s.tmu.edu.tw (Y.-R.L.); 10462@s.tmu.edu.tw (H.-C.C.); 2 Department of Orthopedic Surgery, Shuang Ho Hospital, Taipei Medical University, Taipei 23561, Taiwan; active9102078@gmail.com; 3 Division of Plastic Surgery, Department of Surgery, Shuang Ho Hospital, Taipei Medical University, Taipei 23561, Taiwan; 15405@s.tmu.edu.tw; 4 Department of Surgery, School of Medicine, Taipei Medical University, Taipei 11031, Taiwan; 5 Department of Physical Medicine and Rehabilitation, Wan Fang Hospital, Taipei Medical University, Taipei 11031, Taiwan; chang2773@gmail.com; 6 Institute of Injury Prevention and Control, Taipei Medical University, Taipei 11031, Taiwan; 7 Department of Physical Medicine and Rehabilitation, School of Medicine, College of Medicine, Taipei Medical University, Taipei 11031, Taiwan; 8 Department of Rehabilitation and Movement Science, College of Nursing and Health Sciences, University of Vermont, Burlington, VT 05401, USA; escorpizo.reuben@gmail.com; 9 Swiss Paraplegic Research, 6207 Nottwil, Switzerland

**Keywords:** ICF core sets, burns, rehabilitation, Taiwan

## Abstract

Burn injuries cause disability and functional limitations in daily living. In a 2015 fire explosion in Taiwan, 499 young people sustained burn injuries. The construction of an effective and comprehensive rehabilitation program that enables patients to regain their previous function is imperative. The International Classification of Functioning, Disability, and Health (ICF) includes multiple dimensions that can contribute to meeting this goal. An ICF core set was developed in this study for Taiwanese patients with burns. A consensus process using three rounds of the Delphi technique was employed. A multidisciplinary team of 30 experts from various institutions was formed. The questionnaire used in this study comprised 162 ICF second-level categories relevant to burn injuries. A 5-point Likert scale was used, and participants assigned a weight to the effect of each category on daily activities after burns. The consensus among ratings was assessed using Spearman’s ρ and semi-interquartile range indices. The core set for post-acute SCI was developed from categories that attained a mean score of ≥4.0 in the third round of the Delphi exercise. The core ICF set contained 68 categories. Of these, 19 comprised the component of body functions, 5 comprised body structures, 37 comprised activities and participation, and 7 comprised environmental factors. This preliminary core set offers a comprehensive system for disability assessment and verification following burn injury. The core set provides information for effective rehabilitation strategy setting for patients with burns. Further feasibility and validation studies are required in the future.

## 1. Introduction

On 27 June 2015, in New Taipei City, Taiwan, a color dust explosion caused a massive fire over a large crowd, which resulted in burns to an average of 44% of the total body surface area (TBSA) of 499 young people (mostly aged between 18 and 29 years), 248 of whom exhibited severe burn injuries (burn TBSA > 40%). Moreover, 449 people were admitted to the hospital, of whom 248 were admitted to intensive care units [1]. That fire was the worst incident of massive injury to ever occur in New Taipei City and the most serious public safety accident in recent years. The incident resulted in the deaths of 15 people, yielding a total survival rate of 96.9%. The high survival rate has placed the focus on burn rehabilitation after treatment of acute injuries. One year after the accident, all patients had been discharged from the hospital; 185 patients returned to their daily lives, and 139 patients continued to attend regular rehabilitation programs at outpatient clinics [2,3].

The 2004 Global Burden of Diseases report by the World Health Organization (WHO) stated that approximately 11 million people per year incur burn injuries and that it is the fourth most common substantial injury. This highlights the need for medical resources for complications after burn injury. After acute burn injury treatment, patients with nonfatal chronic burns may experience prolonged hospitalization and sequela such as disfiguring scar formation, joint stiffness, depression, and difficulties in social interaction, making further long-term rehabilitation challenging. Rehabilitation following burn injury includes physical, psychological, and social dimensions [4,5]. To date, several measures have been used to assess outcomes of burn injury, including burn-specific or generic measures. However, some problems remain, such as a lack of consensus regarding which domains to assess, the complexity of the measures, and risk of repetition if using multiple scales [6]. Therefore, the development of a comprehensive tool to assess the health condition of people with post-burn injuries for effective rehabilitation intervention is essential.

The International Classification of Functioning, Disability, and Health (ICF) model, developed by the WHO in 2001, provides a universal classification for an individual’s functioning and disability. The model facilitates international comparison of disability-related data under the biopsychosocial framework with components of body function, body structure, activity and participation, environmental factors, and individual factors. Initially, the main indication for utilizing the ICF and core sets is the functioning aspect [7]. However, neither the ICF categories nor the core sets were evaluated for their diagnostic validity and/or reliability; particularly, the quantitative qualifiers are still arbitrary scales. Nevertheless, determination and identification of the most common deficits in a specific disease or injury is a useful approach to define relevant outcome domains in trials of rehabilitation medicine. Therefore, ICF core sets were generated for application in treating specific diseases and medical problems, for treating a disease in specific stages, for specific medical professionals, and for caregivers or patients themselves. These were established through the selection of priority categories that meet the minimum requirements for evaluating a particular patient population.

Although the ICF provides a platform for disease classification and a systemic approach to identify health-related problems and conditions, it includes more than 1450 categories, hindering effective clinical application [8]. More precise ICF categories describing the disability status of specific patient types are required [9]. Moreover, a comprehensive assessment that enables biopsychosocial evaluation of the overall functional status of patients with chronic burns through a multidimensional approach is lacking. Therefore, we employed the Delphi method to develop an ICF core set specific to chronic burn injury by recruiting professionals in various fields who were experts in caring for patients with burns.

## 2. Materials and Methods

A multidisciplinary team participated in the formal decision-making and consensus process, which included three rounds of the Delphi technique, from 15 November 2015, to 15 July 2016. Professionals or opinion leaders from various fields invited to participate in this study included the following: physicians specializing in plastic surgery, dermatology, rehabilitation, and psychiatry; physical, occupational, and speech therapists; clinical psychologists; social workers; scholars in public health; nurses working in wound care; and the director of the Taiwan Vocational Rehabilitation Association. All of these experts had at least 5 years of clinical or practical experience of interacting with patients with burns. This study was approved by the Joint Institutional Review Board of Taipei Medical University (N201205042).

To avoid missing any items relevant to burn injuries, a comprehensive list of ICF categories was formed by reviewing published studies that focused on the physical and functional evaluation of patients with burns. Articles concerning rehabilitation and burns were searched using the keywords “burns” and “rehabilitation”. All relevant articles in English were included for further evaluation, and quality assessment was performed. The selected articles were then reviewed independently by two reviewers (Wang and Lin), who selected the burn rehabilitation-related factors. In case of conflict of opinion among the reviewers, a third reviewer (Liou) judged and determined whether the factors should be included. All relevant factors were then defined and linked to burn rehabilitation categories, based on systematic and standardized procedures. Then, the list was finalized after discussion among several core authors of this study. Through this process, we selected 162 second-level ICF categories relevant to subacute and chronic burn injuries. In the first Delphi round, we presented the questionnaire containing 162 ICF categories with explanations of the categories and the purpose and process of the study. Participants were able to recommend additional second-level categories that they considered crucial to chronic burn injury during any of the three questionnaire rounds. The questionnaire was sent to each participating expert by post or e-mail. The Round 1 questionnaire contained 44.7% of the second-level ICF categories (162 of 362 categories). Considering availability, only second-level categories were selected for the Delphi process; third and fourth level categories were not discussed. The 162 second-level categories consisted of 40, 20, 69, and 33 items related to body functions, body structure, activities and participation, and environmental factors, respectively. Each category in the questionnaire was accompanied by its WHO ICF Browser description.

During the Delphi process, participants evaluated the importance of each category on a 5-point Likert-type scale, with scores of 5, 4, 3, 2, and 1 indicating that an item was “very important”, “important”, “marginally important”, “unimportant”, and “very unimportant”, respectively. The ratings represented the opinions of experts regarding the relevance of these disabilities or limitations to the health status of people with subacute and chronic burn injury. In developing the second and third round questionnaires, the mean score and standard deviation of each item from all participants in the previous round were displayed; the questionnaire was unique to each expert and indicated their personal score for each item in the previous round. All participants were invited to reconsider their previous grading score and rate each category again in Rounds 2 and 3 with reference to the results from the previous round.

### Data Analysis

A series of data analyses were conducted to develop the ICF core set for subacute and chronic burn injury by using SPSS version 17.0 (IBM, Armonk, NY, USA). First, agreement among experts was assessed by Spearman’s rank correlation (Spearman’s ρ). The level of agreement between each expert’s score and the group mean score (all experts) regarding the importance of each category for the three Delphi rounds was measured using Spearman’s ρ. Moderate and high levels of agreement between individual experts and the group were defined as ρ = 0.3–0.6 and ρ ≥ 0.6, respectively. Second, the semi-interquartile range (SIQR) of the participants’ scores was calculated to grade the consensus for each. The SIQR, a measure of dispersion, is determined by calculating the difference between the upper quartile (the 75th percentile) and the lower quartile (the 25th percentile) of the experts’ scores. An SIQR of ≤0.5 indicated a category with a high level of agreement among the experts. Finally, the importance of each category was assessed by examining the group mean scores of the experts. Categories with a mean score > 4.0 in the third round constituted the ICF core set.

## 3. Results

**Figure 1:**
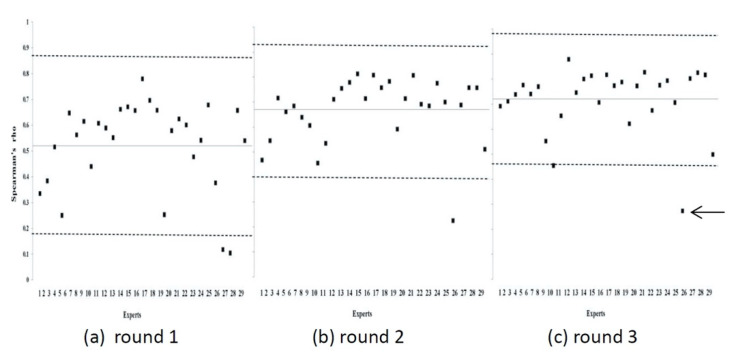
Spearman’s ρ values between each individual expert score and the group mean score of all experts over three rounds of Delphi exercises. The horizontal solid line indicates the mean value of Spearman’s ρ in each round. The area between the dotted lines indicates the mean ±1 standard deviation. Differences between the mean Spearman’s ρ of each round were significant (Wilcoxon signed ranks test, Round 1 vs. Round 2, *p* < 0.001; Round 2 vs. Round 3, *p* < 0.05; Round 1 vs. Round 3, *p* < 0.001). Because Spearman’s ρ for Expert 25 (arrow) was lower than 0.4 in the third round, that data point was regarded as an outlier.

We initially invited 30 experts to participate in this study; however, one person did not complete the three rounds of the Delphi method. The remaining 29 participants completed all three rounds and comprised three plastic surgeons, four physiatrists, one dermatologist, one psychiatrist, two physical therapists, six occupational therapists, one speech therapist, three clinical psychologists, three social workers, two public health scholars, two nurses working in wound care, and the director of the Taiwan Vocational Rehabilitation Association. They were affiliated with different institutions, including six hospitals, one rehabilitation clinic, two universities, and two associations. Their mean period of professional practice was 18.1 years. The experts reached a consensus through the Delphi exercise. The mean Spearman ρ value and 95% confidence interval (CI) were as follows for the first, second, and third rounds: ρ = 0.52 and (95% CI 0.18–0.88), ρ = 0.68 (95% CI 0.40–0.92), and ρ = 0.72 (95% CI 0.46–0.98). Because ρ < 0.4 for participant number 25 in the third round of the Delphi process, it was considered a relative outlier and excluded from the final analysis. Therefore, the group mean score and SIQR were calculated according to the responses of the other 28 experts (Figure 1).

**Figure 2:**
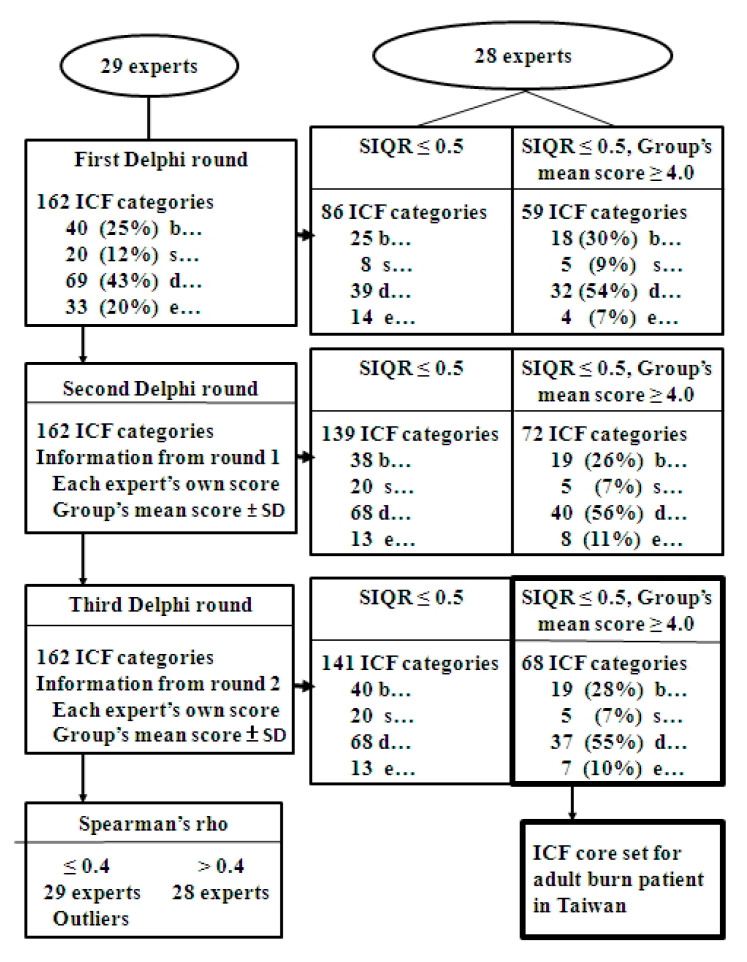
Consensus process of the development of the International Classification of Functioning, Disability, and Health core set for adult patients with burns in Taiwan. The number of selected categories is indicated at each step. The group mean score was the average of all expert scores. Letters b…, s…, d…, and e… indicate the categories of the body functions, body structures, activities and participation, and environmental factors components. SIQR, semi-interquartile range.

Standard SIQR score analysis revealed that 141 items reached a good consensus (SIQR ≤ 0.5). In total, 68 ICF categories had an SIQR of ≤0.5 and a group mean score of ≥4.0. Finally, the ICF core set for subacute and chronic burn injury comprised these 68 ICF categories that achieved a good consensus. The ICF core set contained 68 ICF second-level categories, representing 42% of the original 162 relevant categories in the study (Figure 2).

Of these, 19 categories were from the body functions component, 5 were from the body structures component (Table 1), 37 were from the activities and participation component (Table 2), and 7 were from the environmental factors component (Table 3).

## 4. Discussion

Through three rounds of Delphi exercises to reach a consensus among specialists of multiple disciplines with a focus on burn injuries, a comprehensive ICF core set for chronic burn injuries was developed under the framework of the biopsychosocial model. We identified 68 categories of the ICF core set for post-burn injuries rehabilitation using the Delphi consensus process. Nineteen of them were from the body functions component, 5 were from the body structures component, 37 were from the activities and participation component, and 7 were from the environmental factor component. In addition to providing information regarding body function and body structure, this ICF core set provides information regarding participation and environmental factors. On the basis of the ICF framework, our study developed an ICF core set for burn injuries to facilitate effective clinical practice and rehabilitation.

In the body functions ICF component, selected categories reflected the problems commonly encountered by patients with chronic burns. Most items in the body function category of the core set were related to the skin and related structures (b8 code). The depth of a burn wound and its healing potential are the most crucial determinants of therapeutic management and of residual morbidity or scarring [4,5]. These factors, which affect a patient’s long-term appearance and function, are indicators of prognosis. The findings of the aforementioned studies are reflected in our ICF core set—the three categories of the ICF with the highest scores were from functions of the skin and related structures, namely the protective, reparative, and sensory functions of the skin. In addition to categories concerning the skin and related structures, categories regarding neuromusculoskeletal- and movement-related functions (b7 code) were also critical in the burn ICF core set. Movement restriction is common following burn injury, particularly when joints are involved. Problems related to joint mobility, such as reduced range of motion or contractures and reduced muscle strength, which results from muscle mass loss in severe burns or from periods of immobility due to pain, can all result in long-term functional impairment. Falder et al. in 2009 also listed skin and neuromuscular functions as the first two core domains in burn outcome assessment [9]. A review of ICF-related studies indicated that the role of mental functions (b1 category) was highly valued, with most categories matching existing measurement tools [10,11,12]. These findings are relevant to our study because b1 categories (mental functions) played a crucial role in our ICF core set. However, instead of selecting the categories of consciousness, attention, memory, and cognitive function, which may be impaired during the acute resuscitation stage, our specialists selected sleep, emotions, energy, and drive functions. This may be explained by the fact that our ICF core set is focused on the rehabilitation of patients with chronic burns, which allows them to regain function rather than endure distress during the acute stage of burn injuries.

The activity and participation component counted for more than half of our ICF core set, and half of the categories were derived from chapter d4 “mobility”, followed by d5 “self-care”. For discharged patients with chronic burns returning to daily life, the ability to manage activities of daily living is essential. Our ICF core set emphasizes the importance of mobility and self-care categories for the effective rehabilitation of patients with chronic burns. In addition to self-care and mobility, our core set also included the “interpersonal interactions and relationships” (d7) and “major life areas” (d8) categories. Because of the increasing survival rate of patients with burns, their ability to return to work and rejoin society is becoming more important, specifically in terms of overcoming cosmetic problems, achieving a high level of independence, and rejoining society, ultimately reducing the burden of burn injuries on society.

A review of the measurement tools frequently used in patients with burns revealed that the role of environmental factors has rarely been addressed. In the measurement tool developed in 2011 by Wasaik, 2 out of the 50 items (4%) were environmental factors. Moreover, in the Burns Questionnaire, only 6% of items were related to environmental factors. However, under the conceptual framework of the ICF, environmental factors can play a key role in a person’s activity at home, at work, and in the community, and they must be classifiable to understand their effects on health and function as a reflection of quality of life outcomes [13]. Osborne et al. suggested that environmental factors be addressed in future editions of the Burn Outcomes Questionnaire developed in the Multi-Center Benchmarking Study [14]. In our study, seven categories related to environmental factors were selected, comprising 10.3% of the ICF core set. In the core set developed by Osborne et al., several categories in the environmental factors component were derived from “products and technology” (e1), including products and technology for personal consumption, products for personal use in daily life, transportation, communication, education, employment, buildings, culture, sports and recreation, and assets [15]. In our core set, none of the categories from chapter e1 were selected; instead, we focused on “services, systems, and policies” (e5). In Taiwan, the People with Disabilities Rights Protection Act supports people with disabilities by providing social welfare services, including equipment to assist in transportation and daily living, such as wheelchairs and assistive technology or devices [16]. Furthermore, Taiwan’s National Health Insurance (NHI) system provides comprehensive medical care to residents. The NHI finances health care for all and offers unrestricted access to medical facilities; thus, the considerable expense of acute and chronic care becomes less of a concern for patients. Furthermore, in the case of a public safety event, the government integrates departments to manage the emergent demands for medical professionals and equipment, sets up daytime burn care centers for rehabilitation needs, assists with school-interruption concerns, and provides counseling for employment, which are practical matters that individuals must address when returning to normal life. Therefore, the categories of “health services, systems, and policies” (e580) and “social security services, systems, and policies” (e570) fully cover the categories in chapter e1.

Some studies have attempted to develop ICF core sets to classify and describe functioning, disability, and health in patients with burn injury [15]. Most authors have adopted one of the following methodologies: consulting existing measurement tools frequently used in patients with burns, selecting multiple measurements after a systemic review or identifying burn-specific outcome measurements from single multicenter studies, determining the closest links between individual items and corresponding parts of the ICF, and developing an ICF core set with matched chapters or categories [17,18,19,20]. However, some items cannot be matched to the ICF with the linking method, which limits the comprehensiveness of the outcomes proposed by some studies. Our study collected information reflecting the first-hand experience of specialists in the burn field by using the Delphi method. Our comprehensive multidisciplinary team of burn care experts were able to accurately capture the effects of health conditions of patients with burn injuries under the biopsychosocial framework. Furthermore, through the use of importance scoring by experts, the relevance of each category could be listed on a scale rather than as “yes” or “no”, and categories were selected or matched using the linkage method, which possibly involved subjective judgment bias.

## 5. Limitations

Our study had some limitations that should be noted. First, we identified this core set through Delphi-based consensus with professionals from various burn-related fields. Because the core set was not developed in consultation with patients who incurred burns, its validity and feasibility may be affected. Thus, further validation of this core set is necessary for clinical application in rehabilitation settings. Second, participants’ perceptions of patient burn severity and time to recovery may have varied—some may have reflected on burns during the acute admission period, whereas others may have reflected on the chronic period after patients have returned to the community. To mitigate this discrepancy, we provided a description of the situation of the patients with burn injuries to the experts before they completed the questionnaire. Finally, despite the diverse professional fields and affiliations of the participating experts, the study was limited to Taiwan, and the opinions of professionals in burn-related fields may vary by country. Nevertheless, we believe that our core set is applicable to patients not only in Taiwan but also in other developed countries because the clinical course of burn injuries is similar throughout the world.

## 6. Conclusions

This ICF core set, developed using the Delphi consensus process, provides a multi-dimensional framework for the evaluation of functional health in patients with sequelae of burns. Effective rehabilitation strategies for patients after burns could be designed on the basis of this ICF core set. Further study on the feasibility and validity of this ICF core set for patients with chronic burn injuries is required in the future.

## Figures and Tables

**Table 1:** International Classification of Functioning, Disability, and Health categories of the body functions and body structures components rated as important for the assessment of chronic burn injury over the three rounds of Delphi exercises.

ICF Code	ICF Category Title	Round 1	Round 2	Round 3
Body Functions component				
b130	Energy and drive functions	4.1 (0.8)	4.1 (0.5)	4.1 (0.4)
b134	Sleep functions	4.6 (0.8)	4.5 (0.8)	4.5 (0.7)
b152	Emotional functions	4.3 (0.8)	4.4 (0.6)	4.3 (0.6)
b270	Sensory functions related to temperature and other stimuli	4.5 (0.6)	4.4 (0.6)	4.3 (0.6)
b280	Sensation of pain	4.8 (0.4)	4.7 (0.5)	4.7 (0.6)
b440	Respiratory functions	4.1(0.5)	4.1(0.4)	4.0(0.5)
b455	Exercise tolerance functions	4.6 (0.5)	4.6 (0.5)	4.4 (0.6)
b550	Thermoregulatory functions	4.7 (0.5)	4.7 (0.5)	4.6 (0.5)
b710	Mobility of joint functions	4.7 (0.5)	4.5 (0.6)	4.6 (0.6)
b720	Mobility of bone functions	4.3 (0.8)	4.3 (0.6)	4.4 (0.6)
b730	Muscle power functions	4.7 (0.6)	4.6 (0.6)	4.5 (0.5)
b740	Muscle endurance functions	4.6 (0.8)	4.6 (0.6)	4.4 (0.6)
b770	Gait pattern functions	4.5 (0.6)	4.4 (0.6)	4.3 (0.6)
b810	Protective functions of the skin	4.8 (0.5)	4.8 (0.4)	4.7 (0.4)
b820	Reparative functions of the skin	4.9 (0.5)	4.8 (0.4)	4.9 (0.4)
b830	Other functions of the skin	4.7 (0.6)	4.8 (0.4)	4.9 (0.4)
b840	Sensation related to the skin	4.7 (0.5)	4.8 (0.4)	4.9 (0.3)
b849	Functions of the skin	3.9 (1.3)	4.3 (0.7)	4.4 (0.5)
b850	Functions of hair	4.3 (1)	4.2 (0.7)	4.2 (0.6)
Body Structures component				
s730	Structure of the upper extremities	4.4 (0.8)	4.3 (0.7)	4.3 (0.7)
s750	Structure of the lower extremities	4.4 (0.8)	4.3 (0.8)	4.3 (0.8)
s760	Structure of the trunk	4.1 (0.9)	4.1 (0.9)	4.1 (0.8)
s810	Structure of areas of skin	4.7 (0.5)	4.7 (0.5)	4.7 (0.5)
s820	Structure of skin glands	4.6 (0.6)	4.7 (0.4)	4.7 (0.5)

Note: values are group (28 experts) mean scores (standard deviation); categories with a mean score of ≥4.0 in each round are underlined.

**Table 2:** International Classification of Functioning, Disability, and Health categories of the activities and participation component rated as important for the assessment of chronic burn injury over the three rounds of Delphi exercises.

ICF Code	ICF Category Title	Round 1	Round 2	Round 3
d230	Completing a daily routine	4.2 (1)	4.3 (0.7)	4.3 (0.5)
d240	Coping with stress and other psychological demands	4.3 (1)	4.5 (0.6)	4.4 (0.6)
d410	Changing basic body position	4.6 (0.7)	4.6 (0.6)	4.5 (0.6)
d415	Maintaining a body position	4.5 (1)	4.6 (0.6)	4.6 (0.6)
d420	Independently moving from one place to another	4.6 (0.8)	4.5 (0.6)	4.6 (0.6)
d429	Changing and maintaining body position, other specified and unspecified	4.3 (1)	4.3 (0.6)	4.1 (0.6)
d430	Lifting and carrying objects	4.5 (0.8)	4.4 (0.6)	4.4 (0.6)
d435	Moving objects with the lower extremities	4.3 (1.1)	4.2 (0.6)	4.3 (0.6)
d440	Precise hand movement	4.7 (0.6)	4.7 (0.5)	4.7 (0.4)
d445	Hand and arm use	4.7 (0.5)	4.8 (0.4)	4.8 (0.4)
d446	Precise foot movement	4.2 (1.1)	4.3 (0.7)	4.3 (0.7)
d449	Carrying, moving, and handling objects, other specified and unspecified	4.2 (0.8)	4.2 (0.6)	4.1 (0.5)
d450	Walking	4.8 (0.4)	4.8 (0.4)	4.8 (0.4)
d455	Moving around	4.5 (0.8)	4.7 (0.5)	4.6 (0.6)
d460	Moving around in different locations	4.5 (0.7)	4.5 (0.5)	4.4 (0.6)
d465	Moving around using assistive equipment	4.3 (0.9)	4.2 (0.6)	4.2 (0.6)
d469	Walking and moving, other specified and unspecified	4 (1.1)	4.1 (0.6)	4 (0.6)
d470	Using transportation	4.4 (0.6)	4.4 (0.5)	4.4 (0.6)
d489	Moving around using transportation, other specified and unspecified	4 (0.9)	4.1 (0.6)	4 (0.4)
d510	Washing oneself	4.7 (0.6)	4.8 (0.4)	4.8 (0.4)
d520	Caring for body parts	4.5 (0.7)	4.7 (0.5)	4.6 (0.5)
d530	Using the toilet	4.6 (0.7)	4.8 (0.4)	4.7 (0.4)
d540	Getting dressed	4.7 (0.6)	4.7 (0.5)	4.6 (0.5)
d550	Eating	4.7 (0.5)	4.8 (0.4)	4.7 (0.4)
d560	Drinking	4.7 (0.5)	4.7 (0.5)	4.7 (0.6)
d570	Monitoring one’s health	4.6 (0.5)	4.7 (0.5)	4.7 (0.4)
d598	Self-care, other specified	4.4 (0.7)	4.4 (0.6)	4.4 (0.6)
d710	Basic interpersonal interactions	4.3 (0.7)	4.5 (0.6)	4.5 (0.6)
d720	Complex interpersonal interactions	4.1 (0.9)	4.1 (0.7)	4.1 (0.6)
d740	Formal relationships	4.1 (0.9)	4.3 (0.6)	4.3 (0.6)
d760	Family relationships	4.5 (0.8)	4.4 (0.6)	4.4 (0.5)
d770	Intimate relationships	4.5 (0.7)	4.5 (0.6)	4.4 (0.6)
d825	Vocational training	4.6 (0.8)	4.6 (0.6)	4.5 (0.5)
d845	Acquiring, keeping, and terminating a job	4.4 (0.8)	4.5 (0.6)	4.5 (0.5)
d850	Remunerative employment	4.4 (0.7)	4.5 (0.5)	4.5 (0.5)
d870	Economic self-sufficiency	4.3 (0.9)	4.4 (0.5)	4.3 (0.4)
d920	Recreation and leisure	4.3 (0.7)	4.3 (0.7)	4.2 (0.6)

Note: values are group (28 experts) mean scores (standard deviation); categories with a mean score of ≥4.0 in each round are underlined.

**Table 3:** International Classification of Functioning, Disability, and Health categories of the environmental factors component rated as important for the assessment of chronic burn injury over the three rounds of Delphi exercises.

ICF Code	ICF Category Title	Round 1	Round 2	Round 3
e320	Friends	4 (1.2)	4.1 (0.7)	4 (0.7)
e355	Health professionals	4.1 (1)	4.1 (0.7)	4.1 (0.5)
e410	Individual attitudes of immediate family members	3.9 (1)	4.1 (0.6)	4.1 (0.6)
e460	Societal attitudes	4.2 (1)	4.2 (0.7)	4.2 (0.6)
e570	Social security services, systems, and policies	4.3 (0.7)	4.3 (0.7)	4.3 (0.5)
e580	Health services, systems, and policies	4.4 (0.8)	4.5 (0.5)	4.5 (0.5)
e590	Labor and employment services, systems, and policies	4.3 (1)	4.6 (0.5)	4.6 (0.5)

Note: values are group (28 experts) mean scores (standard deviation); categories with a mean score of ≥4.0 in each round are underlined.

## Data Availability

Data available on request due to restrictions eg privacy or ethical.
